# Irisin Is Correlated with Blood Pressure in Obstructive Sleep Apnea Patients

**DOI:** 10.1155/2021/4717349

**Published:** 2021-11-11

**Authors:** Xing Wang, Zhengjiao Zhang, Xiaoxin Lan, Keyou Fu, Guanhua Xu, Jingyi Zhao, Haibo Yuan

**Affiliations:** ^1^Department of Respiratory Medicine and Sleep Center, First Hospital of Jilin University, Changchun 130021, China; ^2^Department of Neurology and Sleep Center, People's Hospital of Jilin Province, Changchun, China

## Abstract

**Background:**

Despite approximately 95% primary cases of hypertension, secondary hypertension seems to be common with resistant forms. Notably, obstructive sleep apnea (OSA) is known as a common cause of secondary hypertension and has a major characteristic of obesity. Irisin acts as a link between muscles and adipose tissues in obesity, playing an essential role in human blood pressure (BP) regulation. However, whether irisin is associated with secondary hypertension caused by OSA and how it takes effect essentially have not been elucidated.

**Purpose:**

To investigate the changes of irisin and its relationship with BP in OSA.

**Methods:**

72 snoring patients finished Epworth Sleep Scale (ESS) evaluation before polysomnography (PSG). BP was the average of three brachial BP values by mercury sphygmomanometer. Serum irisin level was determined by enzyme-linked immunosorbent assay (ELISA). Results were analyzed by SPSS software.

**Results:**

Irisin was higher in the severe and quite severe group than that in control and nonsevere groups (*p* < 0.05). For BP, significant differences were found between the control group and the other three groups (*p* < 0.05) and between the quite severe and the other three groups (*p* ≤ 0.001). Positive correlations were found between irisin and apnea-hypopnea index (AHI), AHI and BP, and irisin level and BP. Negative correlations were between irisin and SpO_2_ nadir and SpO_2_ nadir and BP. Positive correlation still existed between AHI and irisin even after adjusting for some obesity-related variables.

**Conclusions:**

Irisin may serve as a potential biomarker for severity of OSA independently of obesity and imply the development of hypertension.

## 1. Introduction

Nowadays, OSA tends to be a growing health concern, and the estimated prevalence ranges from 2% to 14% worldwide [1, 2]. OSA is characterized by recurrent episodes of partial (hypopnea) or complete interruption (apnea) in breathing during sleep due to the airway collapse in the pharyngeal region; OSA and its cardiovascular consequences have been widely explored in observational and prospective studies. Most evidence verifies that OSA is an independent cardiovascular risk factor which is associated with obesity, insulin resistance, hypertension, arrhythmias, stroke, coronary artery disease, and heart failure [3]. OSA-related cardiovascular comorbidities are major concerns for prognosis and the complexity of OSA integrated care [4].

Among them, we put more emphasis on hypertension. It has been widely accepted that there exists a bidirectional association between OSA and hypertension. An increasing number of patients with hypertension suffer from OSA, and meanwhile OSA also exerts an obvious impact on the 24-hour BP circadian pattern related to clinical or subclinical organ damages by hypertension [5, 6]. However, the relevant mechanisms of OSA on the proceeding of hypertension have not already been clarified. Continuous positive airway pressure (CPAP), which is the most universally recommended therapy for OSA, is highly effective in improving symptoms but has demonstrated a concordant but limited effect on cardiovascular comorbidities, to be specific, hypertension [5, 7, 8]. Therefore, deciphering the molecular pathways involved in OSA-related adverse cardiovascular consequences is the priority, which may make new pharmacological targets available to improve OSA severity and reduce BP, in combination with or as an alternative to CPAP.

Many researches have shown that a great number of cytokines are involved in the occurrence and development of OSA [9]. Surprisingly, irisin, an exercise-derived myokine and an important regulator of energy metabolism, has been found to be involved in the regulation of BP [10–12]. Significantly, subjects of these studies are all obese patients. However, OSA is almost twice as common in obese than in normal-weight adults. A 10% weight gain gives rise to a 32% increase in the AHI; a 10% reduction yields a 26% improvement in OSA severity [13]. In addition, some investigators suppose that altered irisin/BDNF axis may be causative for circadian misalignment which leads to the excessive daytime sleepiness in OSA [14]. Moreover, notably some studies have also shown that serum irisin concentrations are still closely linked with the prevalence of OSA after getting rid of obesity interference by adjusting for BMI [15]. In a word, these studies tend to claim that OSA may be inextricably bound up with the changes of irisin levels.

Nevertheless, there remains some controversy whether irisin could promote or prevent the development of hypertension in OSA patients and there were some divergences on the changing trend of irisin levels during the progression of OSA. Therefore, in order to reveal the feature of irisin concentration changes produced by OSA and the impact on the risk of BP, we designed a case-control study to investigate it thoroughly.

## 2. Method

### 2.1. Subjects

A total of 72 participants were recruited from those patients who sought medical advice directly or indirectly from the sleep center in the First Hospital of Jilin University with the suspicious diagnosis of OSA. OSA was diagnosed according to the relevant clinical practice guidelines by American Academy of Sleep Medicine (AASM) via overnight polysomnography [16].

All subjects were asked to complete a questionnaire to explore the prior illnesses and drug utilization. We excluded patients who suffered from any infectious disease, cancer, coagulation disorder, and chronic disease including chronic obstructive pulmonary disease, diabetes mellitus, liver cirrhosis, thyroid dysfunction, rheumatoid arthritis, chronic renal failure, psychiatric disorders, and/or sleep disorders other than OSA (upper airway resistance syndrome or narcolepsy). Active use of medications based on physician-adjudicated conditions like vitamins, hormones, immunosuppressants, or free radical scavengers was also the exclusion criteria for this study. The exclusion criteria and assessment protocols were identical for all the studied groups.

The study was approved by the Ethics Committee of the First Hospital of Jilin University. All the participants included in the study signed written informed consent/assent forms.

### 2.2. Anthropometric Variables

All consecutive participants underwent the detailed physical examination and baseline anthropometric measurement in our sleep center, including weight, height, neck circumference (NC), waist circumference (WC), and hip circumference (HC). Body habitus was measured with subjects in light clothing without shoes using standard anthropometric methods. They were all the means of three repetitive measurements.

In detail, weight was measured on a balance beam. WC was measured at the level midway between the lower rib margin and the iliac crest at the end of normal expiration. NC was measured with people's head erect and eye facing forward, horizontally at the upper margin of the laryngeal prominence (Adam's apple) [17, 18]. HC was measured at the maximum protuberance of the buttocks. These three kinds were exactly measured to the nearest centimeter using inextensible tape. Additionally, BMI (kg/m^2^) was calculated using the formula as follows: body weight (kg) divided by the square of body height (m^2^).

Physical activity (PA) was assessed using the self-administered last seven-day recall short version of the International Physical Activity Questionnaire (IPAQ). The amount of PA was expressed in standard metabolic equivalent units (METs) as multiples of resting metabolic rate by minutes of performance during a week [19–21].

### 2.3. The Epworth Sleepiness Scale (ESS)

ESS was completed prior to PSG to assess daytime sleepiness. It was recorded through an eight-item self-administered score from 0 to 24, and a higher score reflects a higher level of daytime sleepiness.

### 2.4. BP Levels

Systolic blood pressure (SBP) and diastolic blood pressure (DBP) levels were assessed in the right arm at the heart level with the patients in the supine position after remaining seated for 5 min of rest in a quiet clinic. BP values were obtained through three systolic and diastolic blood pressure readings taken by Mercury sphygmomanometer. If BP measurements varied by 10 mmHg, an additional measurement was performed. The accumulated measurements were then averaged to determine overall SBP and DBP used for the final analysis. All participants were examined and evaluated by the same researcher.

### 2.5. Sleep Studies and Polysomnography

Polysomnography was performed for all subjects by Polysomnographic Recording Systems (Alice 5, Philips Respironics, Murrysville, PA, USA, and SOMNOscreen™ plus, SOMNOmedics GmbH, Germany) between 22:00 and 06:00. Oronasal flow, thoracoabdominal movements, electrocardiography, submental and pretibial electromyography, electrooculography, electroencephalography, and transcutaneous measurement of arterial oxygen saturation were recorded, and criteria of AASM were used for scoring the sleep stages. We extracted the SpO_2_ nadir from the source. Apnea was defined by the absence of more than or equal to 90% airflow from baseline for over 10 s. Hypopnea was defined as the decrease which is more than or equal to 30% airflow lasting for over 10 s resulting in arousal or oxygen desaturation, a 3% or greater decrease in oxyhemoglobin saturation.

AHI was calculated as the number of apneas and hypopneas per hour during sleep time. Owing to the high proportion of severe and quite severe patients visiting our sleep center, according to the traditional classification method the number of patients in severe group did not match up with that in other groups. Hence, we adjusted the division standard to balance the number of groups better and made it beneficial to the result analysis. The patients were divided into four groups according to AHI: control group (AHI < 5 N/h), nonsevere (AHI 5.0–29.9 N/h), severe (AHI 30–59.9 N/h), and quite severe (AHI ≥ 60 N/h) [17].

### 2.6. ELISA Measurement for Serum Irisin

After overnight polysomnography monitoring, fasting blood samples for each subject were taken in the morning and collected into centrifuge tubes. The samples were centrifuged within 2 h at 1500 rpm for 10 min at 4°C. The obtained plasma was stored at −80°C and was thawed before use. The irisin levels in plasma were measured by using commercially available ELISA kits (CUSABIO, USA), and the experiments were carried out based on the manufacturers' instructions. All the samples were tested in duplicate, and the average values were computed. For methodology validation, we evaluated the intra-assay and interassay variability, which were less than 15% for irisin measurement.

### 2.7. Statistical Analysis

Subjects' baseline characteristics and serum irisin levels were presented as means ± standard deviations or means with 95% confidence intervals (CIs). Normal distribution was assessed through Shapiro–Wilk test. For the statistical comparisons among four groups, one-way analysis of variance (ANOVA) followed by post hoc contrast for numerical variables and Bonferroni was performed to evaluate the significance of difference between two groups. Correlations between variables and polysomnography parameters were sought by way of the Spearman correlation test. The Spearman correlation coefficients (rho) were used to quantify the correlations between two variables. The statistical analysis was performed with the aid of social sciences (SPSS) software version 23.0. For 95% confidence interval, a two-tailed *p* value <0.05 was considered to be statistically significant.

## 3. Results

### 3.1. General Characteristics

The demographic and clinical characteristics of all 72 subjects are shown in Table 1; age ranges from 22 to 67 years. BMI values were between 18.1 and 38.0 kg/m^2^. As shown in Table 1, none of heights differed markedly between the groups (all *p* > 0.05). All of weight, BMI, NC, WC, HC, WC/HC, SpO_2_ nadir, ESS, SBP, and DBP levels differed significantly between the groups (all *p* ≤ 0.001).

Specifically, NC, WC/HC, BMI, and weight were significantly higher in severe and quite severe OSA groups than those in control group (all *p* ≤ 0.001), and SPO_2_ nadir was lower in the three OSA groups than that in control group. SBP and DBP in quite severe groups are significantly higher than those in the other three groups (all *p* ≤ 0.001). ESS score was higher in the two severe OSA groups than that in control group and nonsevere group (*p* < 0.05). There was no significant difference for physical activity level among all the groups (*p* > 0.05). More detailed data comparison and analysis are shown in Table 1.

### 3.2. Serum Irisin Levels

**Figure 1:**
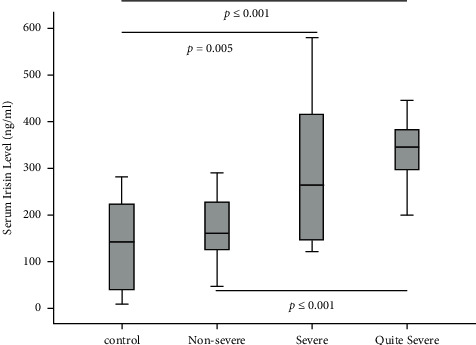
Serum irisin level in control group and the three OSA groups.

Serum irisin level was higher in quite severe OSA group (345.74 ± 99.53 ng/ml) than that in control group (140.29 ± 97.72 ng/ml) and that in nonsevere group (201.13 ± 123.91 ng/ml) (control: *p* ≤ 0.001; nonsevere: *p* ≤ 0.001), and it was also higher in severe group (287.17 ± 143.52 ng/ml) than that in control group (*p* < 0.01). There was no significant difference found between severe group and quite severe group (Figure 1).

### 3.3. Correlation between Serum Irisin Level and AHI

**Figure 2:**
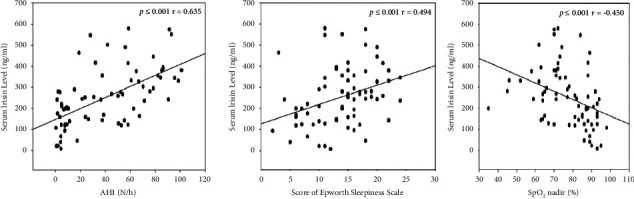
Correlation between serum irisin level and ESS, AHI, and SpO_2_ nadir.

By analyzing the total population of all snoring subjects (*n* = 72), we found that serum irisin level was positively correlated with AHI (*p* ≤ 0.001, *r* = 0.635). In addition, serum irisin levels were correlated positively with ESS and negatively with SpO_2_ nadir, respectively (*p* ≤ 0.001, *r* = 0.406; *p* ≤ 0.001, *r* = −0.450) (Table 2 and Figure 2). No correlation was found between physical activity level and serum irisin level (*p*=0.289, *r* = 0.127).

BMI, NC, and WC/HC were also associated with serum irisin level. When adjusted for these factors, serum irisin level is still positively correlated with AHI (BMI controlled: *p*=0.044, *r* = 0240; NC controlled: *p* ≤ 0.001, *r* = 0.419; WC/HC controlled: *p*=0.026, *r* = 0.280). Considering the significant difference of age among groups (*p*=0.012), we adjusted the factor of age and still found the important difference between serum irisin level and AHI (age controlled: *p* ≤ 0.001, *r* = 0.584).

### 3.4. Correlation between Serum Irisin Level and the Level of BP

**Figure 3:**
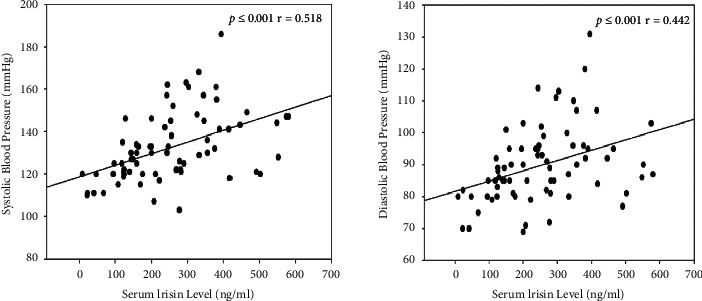
Correlation between blood pressure (SBP and DBP) and serum irisin level.

As was shown in Table 2 and Figure 3, a significant positive correlation was found between BP and irisin (systolic pressure: *p* ≤ 0.001, *r* = 0.518; diastolic pressure: *p* ≤ 0.001, *r* = 0.442), suggesting that serum irisin levels were associated with the increasing BP. Additionally, both ESS score and SpO_2_ nadir correlated significantly with the BP (ESS: systolic pressure: *p*=0.007, *r* = 0.318; diastolic pressure: *p*=0.007, *r* = 0.313; SpO_2_ nadir: systolic pressure: *p* ≤ 0.001, *r* = −0.562; diastolic pressure: *p* ≤ 0.001, *r* = −0.506) (Table 2 and Figure 3).

## 4. Discussion

Recent studies have focused on the roles of serum irisin level in OSA [22, 23]. In this study, we found that serum irisin level was correlated significantly with AHI, even after adjustment of BMI, WC/HC, and NC. Considering that a great number of patients in our center were severe, the traditional classification of OSA severity was no longer applicable for a reasonable proportion among groups. Additionally, our results revealed the remarkable difference between the quite severe and severe group in AHI, SpO_2_ nadir, SBP, and DBP (Table 1). Without the new classifying approach, we would not have deep insight into the accumulation of pathophysiological changes, the evolution of the disease, and even the formulation of the treatment plan targeted at different disease stratification.

To begin with, many researches have showed that irisin, a hormone-like myokine, is increased in individuals engaged in exercise-induced activities and gradually reduced in those who exercise less actively [24, 25]. However, OSA patients hardly ever do exercise due to obesity, common daytime symptoms including excessive daytime sleepiness and fatigue, or otherwise. We also chose individuals with relatively less exercise as control group. In our study, all subjects have a physical activity level of less than 3000 MET-min/week. As a result, there were no marked difference of PA within groups and no obvious correlation between PA and irisin.

Then, it should be noticed that altered irisin/BDNF axis may be causative for circadian misalignment in OSA, and thus it might be suggested that excessive daytime sleepiness could be a result of the deterioration of circadian rhythm along with changes of serum irisin level [14]. Significant changes in ESS score, a simple questionnaire for patients' self-assessment of daytime sleepiness, were experienced if serum irisin level changed by 1 ng/mL, given the limited serum level of BDNF [26, 27]. In our study, obvious correlations between ESS score and AHI and ESS score and serum irisin level were found (Table 2 and Figure 2). This can partly confirm the elevated irisin level as the severity of OSA increases. Moreover, obesity may be a bridge linking AHI and irisin. Our study illustrates that irisin was frequently associated with BMI, weight, WC/HC, and NC and AHI was tightly connected with BMI (Table 2 and Figure 2). Other studies pointed out that an increase in the body fat mass led to an increase in irisin levels and weight loss after an energy restriction treatment and subsequent weight regain have been found to be related to the decrease and increase in circulating irisin levels, respectively [28, 29]. It is acknowledged that muscle tissue may be closely associated with changes in irisin levels after exercise training whereas in physiopathological like atypical BMI situations such as obesity, adipose tissue would be responsible for the described high irisin levels and shared stronger bonds with irisin regulation than other tissues [30]. For one thing, it could be envisioned that increased processing and release of muscle irisin induced by signals originating in adipose tissue resulted in the increased irisin in obesity [31]. For another, a previous study also demonstrated that the adipose tissue secretion could provide circulating FNDC5/irisin, which means that irisin is also produced by adipose tissue directly especially in obese patients [32]. Increased irisin level has been proposed to be the result of the development of irisin resistance and serves as an adaptive response that compensates for the decreasing insulin sensitivity and metabolic disturbances associated with obesity [33]. Additionally, after CPAP (continuous positive airway pressure) treatment, adiponectin levels and leptin levels decreased significantly in obese patients [34, 35]. Notably, leptin, adiponectin, and irisin were metabolic hormones with a similar structure and function of modulating muscle and bone metabolism. They were also mostly considered as potential biomarkers for insulin sensitivity and closely associated with obesity and OSA [35–37], which may change consistently in OSA but still remain to be studied further.

As was mentioned above, in the obese or overweight state, the massive secretion directly or indirectly by adipocytes was mainly responsible for the considerable irisin increase. However, under nonobese conditions, the muscle cells take the lead in regulating the high level of circulating irisin [13]. It may explain our results that after adjusting for some anthropometric variables on obesity including BMI, NC, and WC/HC, the irisin level remained independently correlated with AHI. Also, some immunohistochemical analysis showed that irisin was located extracellularly between muscle fibers [38]. Nevertheless, whether the increased levels of irisin are the result of raised secretion from muscle cells or of release after muscle damage remains unknown. As for the molecular mechanism, besides the PGC1*α* upregulation that stimulates FNDC5 expression, the deprivation of intracellular muscle ATP after exercise might trigger synthesis of FNDC5 and release of irisin [31]. In addition, recently some study contradicted our results [15], which shows the negative correlation between serum irisin concentration and AHI by a BMI and physical activity-matched study (*r* = −0.787, *p* ≤ 0.001). It is noted that the BMI of their OSA group ranges from 21.8 to 25.6. Nevertheless, obesity is the most important risk factor for OSA; at least 70% of patients are obese [39]. Hence, we doubt whether the results remain the same on condition that BMI increases a lot.

Meanwhile, it could be discovered in our results that SpO_2_ nadir was related to AHI and irisin, which means that SpO_2_ nadir may be regarded as intermediate pathway to connect AHI and irisin. It is reported that irisin expression is triggered by hypoxia-induced sympathetic activation and involved in activation of inflammatory cascades in various organs [40–42]. Also, some previous studies showed that irisin functioned as a protective factor by upregulating the cellular antioxidants, sustaining mitochondrial membrane potential, and reversing ATP production to counter the free radical damage induced by hypoxia [43–45]. As for whether irisin is still related to hypoxic parameters like SpO_2_ nadir or hypoxic burden after adjustments of obesity-related variables has yet to be investigated deeply. In a word, we have discussed the potential of irisin in OSA and found that irisin could regulate muscle growth [46] and metabolism as a compensatory response during the development of OSA [38, 47, 48]. The mentioned molecular, biochemical, and metabolic data on the effect of irisin all conduced to understand the nature and feature of irisin. However, the role of irisin in obese patients with OSA as well as its comorbidities is unclear for the time being. And either the muscle or the adipose tissue main functions is still controversial. Thus, further mechanistic research is necessary to support these associations.

Some previous studies indicated that higher circulating irisin levels were related to a variety of cardiovascular diseases (CVD), like vascular atherosclerosis and ischemic stroke, suggesting that elevated circulating irisin levels could be a sign of vascular structural or functional changes and adverse cardiovascular events [49–52]. As no adequate data on irisin function has been unequivocally demonstrated, this hypothesis is still an open question. However, the evidences are the strongest to support that the systemic hypertension was a consequence of severe OSA [53–55]. The underlying mechanisms include the enhanced chemoreceptor sensitivity and overproduction of superoxide ion on resistance vessels [55–57]. Meanwhile, vascular endothelial injury induced by hypoxia might also be considered as an imperative initial factor of hypertension [58, 59]. Furthermore, some studies showed that irisin levels were significantly and positively correlated with systolic [49] or diastolic blood pressure [60] or both of them [13, 49, 61], which consist with our study. Also a significant direct correlation was found between the irisin concentration and the circulating EPCs (endothelial progenitor cells) level [13] which was tightly relevant with hypertension [62]. Briefly, it was estimated that irisin may function as a mediator between OSA and increasing BP.

### 4.1. Problems to Be Solved

Our study has some limitations that should be considered when interpreting the results: first, our cohort sample size was relatively limited and larger study cohorts are needed in the future. Second, BMI was used to assess our study population as a good method but it cannot differentiate between body fat and lean body mass. And the long-term physical activity status of subjects was not evaluated. Finally, many questions have been raised with regard to the molecular regulatory pathways involved in the correlation between irisin level, AHI, and BP. Although whether these influences of OSA are sufficient, independently of obesity, to contribute significantly to the “hypertension” remains unsettled, irisin would still be an attractive tool for treating or intervening hypertension and OSA considering its relationship with human adipocytes, myocytes, and cardiovascular system.

## 5. Conclusion

To summarize, this paper indicates that circulating irisin has the potential implication as a diagnostic biomarker for monitoring and predicting the occurrence and progression of BP increase in OSA patients. However, further studies are required to determine the molecular mechanism of irisin acting on OSA to make it a better therapeutic agent to combat cardiovascular diseases, especially hypertension.

## Figures and Tables

**Table 1:** Demographic characteristics of the subjects in this study.

	Control	Nonsevere OSA	Severe OSA	Quite severe OSA	*p* value
*N*	14	19	18	21	—
Age (years)	33.93 ± 7.74	40.58 ± 8.07	45.44 ± 10.03^*∗*^	42.10 ± 11.38	0.012
Weight (kg)	66.74 ± 8.23	82.54 ± 12.13^*∗*^	86.47 ± 10.00^*∗∗*^	92.89 ± 14.31^*∗∗*^^#^	≤0.001
Height (m)	1.72 ± 0.06	1.75 ± 0.06	1.75 ± 0.05	1.72 ± 0.06	0.221
BMI (kg/cm^2^)	22.62 ± 2.55	26.78 ± 3.05^*∗*^	28.32 ± 2.88^*∗∗*^	31.16 ± 3.60^*∗∗*^^##&^	≤0.001
NC (cm)	37.68 ± 2.44	41.90 ± 3.24^*∗∗*^	42.39 ± 2.17^*∗∗*^	44.24 ± 2.57^*∗∗*^^#^	≤0.001
WC (cm)	89.71 ± 7.22	98.16 ± 7.85^*∗∗*^	102.08 ± 5.91^*∗∗*^	108.98 ± 8.74^*∗∗*^^##&^	≤0.001
HC (cm)	97.18 ± 4.77	103.43 ± 5.23^*∗*^	107.19 ± 3.83^*∗∗*^	111.06 ± 7.41^*∗∗*^^#^	≤0.001
WC/HC	0.87 ± 0.03	0.91 ± 0.02^*∗*^	0.93 ± 0.03^*∗∗*^	0.95 ± 0.03^*∗∗*^^##&^	≤0.001
AHI (events/h)	3.01 ± 1.54	14.86 ± 7.64^*∗*^	46.97 ± 9.56^*∗∗*^^##^	80.57 ± 12.26^*∗∗*^^##&&^	≤0.001
SpO_2_ nadir	91.71 ± 3.50	84.42 ± 6.62^*∗*^	74.56 ± 9.03^*∗∗*^^#^	63.76 ± 11.21^*∗∗*^^##&&^	≤0.001
ESS	10.36 ± 4.22	10.89 ± 5.03	15.11 ± 4.97^*∗*^^#^	16.86 ± 4.97^*∗∗*^^##^	≤0.001
SBP (mmHg)	116.71 ± 7.04	128.58 ± 10.12^*∗*^	131.56 ± 12.62^*∗*^	147.81 ± 15.77^*∗∗*^^##&&^	≤0.001
DBP (mmHg)	79.50 ± 6.61	87.47 ± 8.83^*∗*^	88.28 ± 7.16^*∗*^	100.48 ± 12.68^*∗∗*^^##&&^	≤0.001
PA (MET-min/week)	792.00 ± 248.90	822.29 ± 379.17	965.83 ± 538.12	891.38 ± 643.29	≥0.05

Data are expressed as the mean ± SD or means with 95% confidence intervals. AHI, apnea-hypopnea index; BMI, body mass index; SpO_2_, peripheral capillary oxygen saturation; ESS, Epworth Sleepiness Scale; NC, neck circumference; WC, waist circumference; HC, hip circumference; WC/HC, waist circumference/hip circumference; SBP, systolic BP; DBP, diastolic BP; PA, physical activity; MET: standard metabolic equivalent unit. ^*∗*^*p* < 0.05 vs. the control group; ^*∗∗*^*p* ≤ 0.001 vs. the control group; ^#^*p* < 0.05vs. the nonsevere OSA group; ^##^*p* ≤ 0.001 vs. the nonsevere OSA group; ^&^*p* < 0.05 vs. the severe OSA group; ^&&^*p* ≤ 0.001 vs. the severe OSA group.

**Table 2:** Correlation analysis between different parameters in the OSA patients.

	Irisin (ng/ml)		SBP (mmHg)		DBP (mmHg)		AHI (events/h)	
	*r*	*p* value	*r*	*p* value	*r*	*p* value	*r*	*p* value
BMI	0.652	≤0.001	0.633	≤0.001	0.580	≤0.001	0.722	≤0.001
NC	0.488	≤0.001	0.603	≤0.001	0.532	≤0.001	0.629	≤0.001
WC/HC	0.516	≤0.001	0.497	≤0.001	0.378	≤0.001	0.463	≤0.001
AHI (events/h)	0.635	≤0.001	0.701	≤0.001	0.620	≤0.001	—	—
ESS	0.406	≤0.001	0.318	0.007	0.313	0.007	0.494	≤0.001
SpO_2_ nadir	−0.450	≤0.001	−0.562	≤0.001	−0.506	≤0.001	−0.837	≤0.001
SBP (mmHg)	0.518	≤0.001	—	—	—	—	0.701	≤0.001
DBP (mmHg)	0.442	≤0.001	—	—	—	—	0.620	≤0.001

AHI, apnea-hypopnea index; BMI, body mass index; NC, neck circumference; WC/HC, waist circumference/hip circumference; ESS, Epworth Sleepiness Scale; SpO_2_, peripheral capillary oxygen saturation; SBP, systolic BP; DBP, diastolic BP.

## Data Availability

The original data and Excel data sheet used to support the findings of this study are available from the corresponding author upon request.
